# Complete chloroplast genome of an endangered plant, *Alseodaphne Hainanensis*

**DOI:** 10.1080/23802359.2019.1623731

**Published:** 2019-07-10

**Authors:** Yong Yang, Yu-Kai Chen, Qing Chen

**Affiliations:** a Ministry of Education Key Laboratory for Ecology of Tropical Islands, College of Life Sciences, Hainan Normal University, Haikou, China;; b Bawangling National Nature Reserve, Hainan Province, Changjiang, China

**Keywords:** *Alseodaphne hainanensis*, endangered plant, chloroplast genome, phylogenetic analysis

## Abstract

*Alseodaphne hainanensis* is an endangered species with inhabiting dense forest along valleys, and it is one of the representative species of tropical rain forest in Hainan Island. In this paper, we reported and characterized the complete chloroplast genome sequence of the species assembled from short reads generated by Illumina sequencing. The size of chloroplast was 152,829 bp, a pair of inverted repeats (IRs) separating a large single copy and a small single copy, the size of IRs, LSC and SSC were 20,036 bp, 93,872 bp and 18,885 bp, respectively. A total of 129 genes were predicted, including 81 protein-coding genes, 38 tRNA, 8 rRNA, and 2 pseudogene. Phylogenetic analysis confirmed the position of *A. hainanensis* within the order Laurales.

*Alseodaphne hainanensis* Merrill (Lauraceae) is an evergreen tree which grows to a height of 25 m. It is distributed in parts of Hainan Island and north of Vietnam, inhabiting dense forest along valleys at an altitude of 1400–1700 m. *Alseodaphne hainanensis* is an endangered species and its wood is finely grained, heavy, and durable. The population has been drastically reduced due to long-term utilization and unreasonable logging (Chen et al. 2011). Early studies of *A. hainanense* have mainly focused on the interspecific associations of dominant populations in a community (Chen et al. 2011), wood anatomy (Gao et al. 2009), chemical composition (Chang et al. 2000), and biological characteristics of the plant (Huang et al. 2011). The information of chloroplast genomes has been extensively applied to understanding the evolution history and natural selection process for many plants (Inoue 2011; Zheng et al. 2016). In this paper, we characterized the complete chloroplast genome sequence of *H. hainanensis* to contribute for further phylogenetical and protective studies of this plant.

The plant samples of *A. hainanensis* were collected in Bawangling national nature reserve, Hainan Island (N19°05′49.15, E109°10′27.96) in China, and the specimen was deposited at the botany laboratory of Hainan normal University, Haikou, China. Total genomic DNA was extracted from five mixed fresh leaf tissues using the CTAB method (Doyle 1987). The genome was sequenced on an Illumina HiSeq 2000 platform (Illumina, San Diego, CA, USA) with 150 bp paired-end reads. In total, approximately 10.0 Gb of sequence data were generated.

Using the chloroplast genome of *Alseodaphne gracilis* (NC037489) as reference, the chloroplast genome of *A. hainanensis* was assembled with the program NOVOPlasty (Dierckxsens et al. 2016). The chloroplast genome was annotated with the DOGMA (Wyman et al. 2004) and the circular chloroplast genome map was drawn by OGDRAW.

The complete chloroplast genome of *A. hainanensis* has a typical quadripartite structure with 152,829 bp in length. A pair of inverted repeat (IR) region (20,036 bp) separated the large single copy (LSC) region (93,872 bp) and small single-copy (SSC) region (18,885 bp). The content of GC is 39.12% and chloroplast genome contains 129 predicted genes, including 81 protein-coding genes, 38 tRNA genes, 8 rRNA genes, and 2 pseudogenes. The accession number on GenBank is MK867376.

A phylogenetic analysis was conducted to analyze the position of *A. hainanensis* in the order Laurales, 9 complete chloroplast genome sequence in the order Laurales were aligned by MUSCLE, the sequences were inferred by the maximum likelihood methods with MEGA7 (Kumar et al. 2016), with *Machilus thunbergii* as outgroup. The result shows that *Cinnamomum camphora* in Lauraceae is most closed with *A. hainanensis* (Figure 1). Our result provides an essential DNA molecular data for future conservation genetic studies of this endangered species.

**Figure 1.**
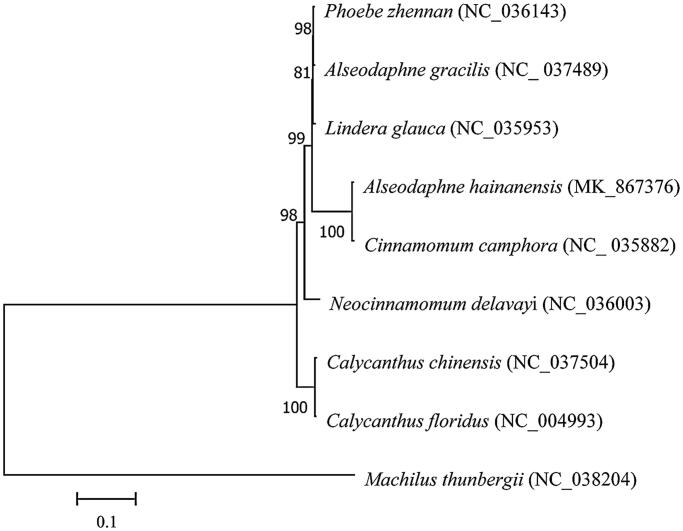
Maximum likelihood tree based on the sequences of 9 complete chloroplast genomes with *Machilus thunbergii* as outgroup. Numbers in the nodes were bootstrap values from 1000 replicates.
